# Expression of Matrix Metalloproteinases and Their Inhibitors in Endometrium: High Levels in Endometriotic Lesions

**DOI:** 10.3390/ijms21082840

**Published:** 2020-04-18

**Authors:** Alice Luddi, Camilla Marrocco, Laura Governini, Bianca Semplici, Valentina Pavone, Stefano Luisi, Felice Petraglia, Paola Piomboni

**Affiliations:** 1 Department of Molecular and Developmental Medicine, Siena University, 53100 Siena, Italy; luddi@unisi.it (A.L.); marrocco@student.unisi.it (C.M.); laura.governini@unisi.it (L.G.); semplici4@student.unisi.it (B.S.); pavalentina13@gmail.com (V.P.); stefano.luisi@unisi.it (S.L.); 2 Department of Experimental and Clinical Biomedical Sciences, University of Florence, 50134 Florence, Italy; felice.petraglia@unifi.it

**Keywords:** endometriosis, ovarian cysts, deep infiltrating endometriosis, metalloproteases, TNF, endometrium

## Abstract

Endometriosis is a condition defined as presence of endometrium outside of the uterine cavity. These endometrial cells are able to attach and invade the peritoneum or ovary, thus forming respectively the deep infiltrating endometriosis (DIE) and the ovarian endometrioma (OMA), the ectopic lesions feature of this pathology. Endometriotic cells display high invasiveness and share some features of malignancy with cancer cells. Indeed, the tissue remodeling underlining lesion formation is achieved by matrix metalloproteinases (MMPs) and their inhibitors. Therefore, these molecules are believed to play a key role in development and pathogenesis of endometriosis. This study investigated the molecular profile of metalloproteinases and their inhibitors in healthy (*n* = 15) and eutopic endometrium (*n* = 19) in OMA (*n* = 10) and DIE (*n* = 9); moreover, we firstly validated the most reliable housekeeping genes allowing accurate gene expression analysis in these tissues. Gene expression, Western blot, and immunofluorescence analysis of MMP2, MMP3, and MMP10 and their tissue inhibitors TIMP1 and TIMP2 demonstrated that these enzymes are finely tuned in these tissues. In OMA lesions, all the investigated MMPs and their inhibitors were significantly increased, while DIE expressed high levels of MMP3. Finally, in vitro TNFα treatment induced a significant upregulation of *MMP3*, *MMP10*, and *TIMP2* in both healthy and eutopic endometrial stromal cells. This study, shedding light on MMP and TIMP expression in endometriosis, confirms that these molecules are altered both in eutopic endometrium and endometriotic lesions. Although further studies are needed, these data may help in understanding the molecular mechanisms involved in the extracellular matrix remodeling, a crucial process for the endometrial physiology.

## 1. Introduction

Endometriosis is a hormone-dependent pathology that affects up to 5–10% reproductive-aged women, and it is characterized by the presence of endometrial tissue in extrauterine locations, primarily at the pelvic level (pelvic peritoneum, ovaries, and rectovaginal septum) and more rarely in other organs and tissues outside the pelvis (colon, liver, lungs) [1]. This disease may appear as lesions of the pelvic peritoneum, as retroperitoneal nodules, and/or lesions on the support structures of the uterus [1]. In the ovary, ovarian lesions are cysts with blood content. These characteristic lesions consist of endometrial stromal cells, endometrial epithelial cells, and extracellular matrix; these cells maintain their receptivity to estrogen undergoing cyclic proliferation and breakdown similarly to the eutopic endometrium. The tissue outbreaks, leading to inflammatory, adherential, and scarring processes that can result in local chronic inflammatory environment that, in turn, further contributes to disease progression [2]. Indeed, endometriosis is considered a pelvic inflammatory condition; in women with endometriosis, peritoneal fluid is characterized by an increased number of activated macrophages and high levels of pro-inflammatory cytokine/chemokine, such as tumor necrosis factor-α (TNFα) and interleukin-8 [3,4,5,6]. The shedding of menstrual endometrial fragments into the peritoneal cavity induces inflammation, and in response to their presence, neutrophils and macrophages are recruited. They are the major source of the elevated proinflammatory and chemotactic cytokines, and the uncontrolled activation of these cells results in persistent and chronic inflammation [7].

Several authors demonstrate an increased level of TNFα in peripheral fluid and endometrium of women with endometriosis and detected a positive correlation between TNFα concentrations and endometriotic lesion size [8,9]. Peritoneal fluid contains higher concentration of proinflammatory and angiogenic cytokines presumably produced from immune cells such as macrophages and from the lesion itself, which contribute to the pathogenesis of endometriosis [10,11].

About the endometriosis origin, several theories were formulated but still none seem to be exhaustive [12]. The most accredited theory is the retrograde menstruation theory, named the Sampson’s theory, proposed in the 1920s and confirmed later [12,13]. This theory explains the physical displacement of endometrial fragments, but additional steps are necessary for the development of endometriotic implants: escape from immune clearance, invasion of surrounding tissues, establishment of local vascularization to obtain growth and survival factors. In fact, more than 90% of women undergo retrograde menstruation; however, the prevalence of endometriosis in the general population is much lower. This discrepancy suggests that women who develop endometriosis are likely to have other genetic factors contributing to development of the disease [14]. In these cells, an alteration in the expression of molecules responsible for the tissue invasion must also be present, for the remodeling of the surrounding matrix, etc. Although endometriosis is a benign disorder, its ability of invasion shares features of malignancy, such as metastatic cancer cells, which overexpress matrix metalloproteases (MMPs) [15,16].

Matrix metalloproteases are a family of zinc-dependent endopeptidases able to degrade the molecules of extracellular matrix (ECM) and basal membrane, and therefore, they are involved in physiological processes that include ECM remodeling, an integral part of tissue growth and differentiation [17]. They are also involved in the processing of several bioactive molecules, such us cytokines and chemokines [18,19,20].

Their activity and functions are regulating by tissue inhibitors of metalloprotease (TIMPs), and it is of paramount importance to maintain the dynamic equilibrium established between the expression of the MMPs and their inhibitors so that the remodeling of the extracellular matrix occurs correctly. Modifications of this balance are related to pathological conditions [12]. Therefore, aberrant regulation of matrix metalloproteases may be the primary cause of endometrial lesion formation in a group of predisposed women. The relationship between endometriosis and MMPs has long been known, and it is widely demonstrated in the literature; in fact, endometrium derived from women with endometriosis shows higher proteolytic activity than endometrium from healthy controls [21,22,23]. However, the studies conducted on human endometriotic tissues are conflicting because of the intervention of numerous variables influencing the results.

Taking into account these observations, the present study assessed the expression of MMP2, MMP3, MMP10, TIMP1, and TIMP2 by quantitative reverse transcription polymerase chain reaction (qRT-PCR) and Western blotting in healthy (HE) and eutopic (EE) endometrium, ovarian endometrioma (OMA), and deep infiltrating endometriosis (DIE) lesions. Furthermore, in vitro expression of selected MMPs and TIMPs in primary human endometrial stromal cells (HESCs) from healthy and eutopic endometrium, after TNFα exposure, was investigated.

## 2. Results

### 2.1. Gene Expression Analysis of MMPs and Their Inhibitors in Healthy and Eutopic Endometrium, OMA, and DIE

In order to clarify the role of metalloproteases and their inhibitors in endometriosis development, *MMP2*, *MMP3*, *MMP10*, *TIMP1*, and *TIMP2* expression was measured in healthy and eutopic endometrium, in OMA and DIE lesions. To this end, endometrial biopsies were collected from patients who underwent surgery for not endometriotic ovarian cysts (healthy, *n* = 15) and from women affected by endometriosis undergoing laparoscopic surgery (eutopic, *n* = 19). At the same time, ovarian endometrioma (OMA, *n* = 10) and deep infiltrating endometriosis (DIE, *n* = 9) lesions were collected.

To perform gene expression analysis, the best reference gene for quantitative expression studies was validated by using geNorm software. This VBA applet ranked the candidate housekeeping according to their stability, from the most to the least stable: *PPIP–TBP–HPRT1–GAPDH–B2M–ACTB.* According to these results, an accurate normalization factor for qRT-PCR data could be calculated by using the three most stably expressed genes, *TBP*, *HPRT1*, and *ACTB*.

**Figure 1:**
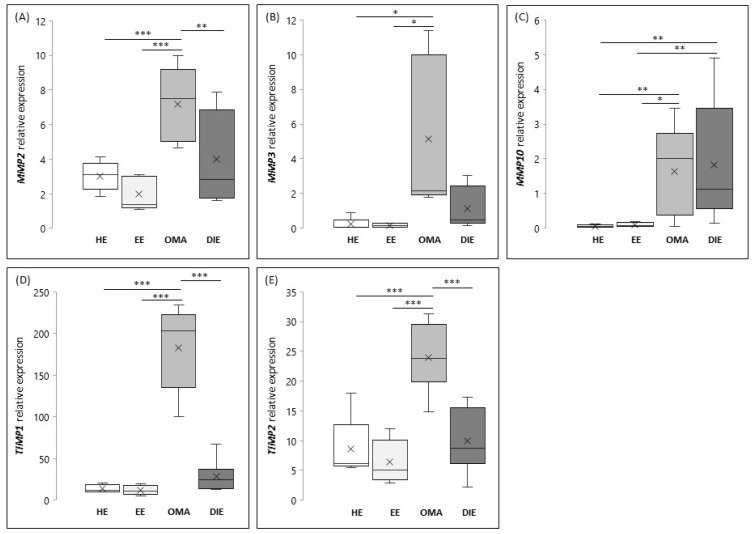
Expression of matrix metalloproteinases (MMPs) and tissue inhibitors of MMP (TIMPs) in human healthy (HE) and eutopic (EE) endometrium, in ovarian endometrioma (OMA) and in deep infiltrating endometriosis (DIE) lesions. MMP2 (**A**), MMP3 (**B**), MMP10 (**C**), TIMP1 (**D**), and TIMP2 (**E**). Graphical diagrams are plotted as box-whisker plots, where boxes show the interquartile range with median and mean values, and whiskers represent min and max confidence intervals. Number of analyzed samples: HE: 15, EE: 19, OMA: 10, DIE: 9. Data represent value obtained by 2^-ΔCt^ method. * *p* < 0.05; ** *p* < 0.01; *** *p* < 0.001.

As shown in Figure 1A, the expression levels of *MMP2* are significantly increased in OMA (*p* < 0.001) when compared to the eutopic and/or healthy endometrium. *MMP2* expression in DIE lesions seems to be more variable, and for this reason, the highlighted increase does not reach the statistical significance when compared to the eutopic endometrium, whereas its expression appears significantly reduced when compared to OMA (*p* < 0.01). As shown in Figure 1B, the expression of *MMP3* is significantly increased in OMA if compared to healthy and eutopic endometrium (*p* < 0.01).

With regard to MMP10 (Figure 1C), we found that the expression of this metalloproteinase is comparable between healthy and eutopic endometrium, while it results significantly increased in endometriotic lesions, both OMA and DIE (*p* < 0.01). Finally, our results demonstrated that tissue inhibitors of metalloproteases are overexpressed in OMA; in fact, mRNA levels of both *TIMP1* and *TIMP2* are significantly increased in OMA when compared to endometrium (healthy and eutopic) or to DIE (*p* < 0.001) (Figure 1D,E, respectively).

Based on this evidence, we further analyzed the correlation between the expression of *MMP2* and *MMP3* with their inhibitors in OMA. Interestingly, we demonstrated that *MMP2* and *TIMPs* (*R* = 0.65, *p* < 0.01, relative to *TIMP1*; *R* = 0.7, *p* < 0.05, relative to *TIMP2*), as well as *MMP3* and *TIMPs* (*R* = 0.65, *p* < 0.01, relative to *TIMP1*; *R* = 0.7, *p* < 0.01, relative to *TIMP2*) were significantly correlated.

### 2.2. Protein Expression and Localization

In order to confirm the data obtained by gene expression analysis, we analyzed protein extracts prepared from endometrium and from endometriotic lesions by Western blot; the overall relative intensity of bands was normalized with respect to β-Actin.

**Figure 2:**
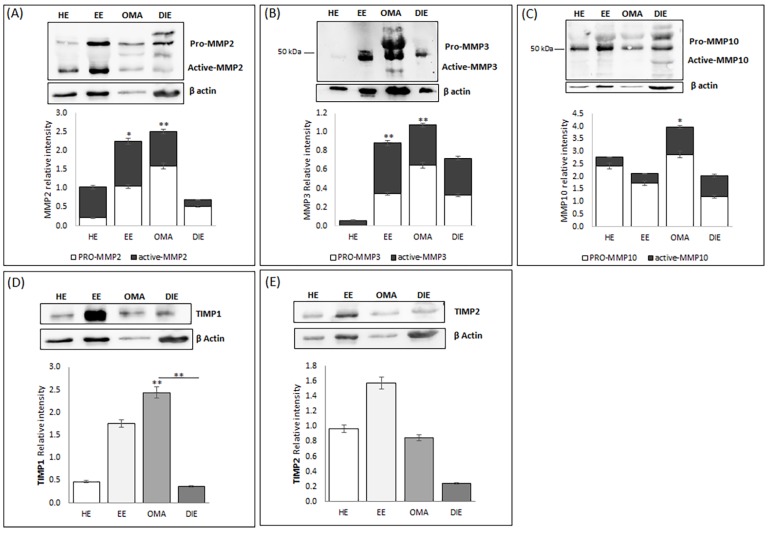
Expression of MMPs and TIMPs in human healthy (HE) and eutopic (EE) endometrium, in endometrioma (OMA) and in deep infiltrating endometriosis lesions (DIE). (**A**–**C**) Western blot of MMP2, MMP3, MMP10; β Actin was immunodetected to control for loading per lane. The relative migrations of pro- and fully mature MMPs are indicated. Computer-assisted semiquantitative analysis of the overall relative intensity of the bands, measured (pixel/mm^2^) and then normalized relative to ACTB. Relative intensities are plotted ratio of active versus pro-form. (**D**,**E**) Western blot of TIMP1 and TIMP2; β Actin was immunodetected to control for loading per lane. The intensity was measured (pixel/mm^2^) and then normalized relative to β Actin. The experiments, independently repeated twice on a pool of 5 patients for each group, gave similar results. * *p* < 0.05; ** *p* < 0.01.

As shown in Figure 2A, Western blot analysis identified several bands corresponding to the predicted molecular weight of pro- and fully mature MMP-2 (indicated at 75–65, and 60 kDa, respectively). Semi-quantitative analysis of the blot demonstrated the presence of increased levels of this metalloproteinase in eutopic endometrium and in OMA, whereas low levels are detectable in DIE; these data may reflect the high interindividual variability already highlighted in DIE tissue by gene expression analysis. Interestingly, while in DIE lesions, MMP2 was mostly present in its pro- form, OMA lesions and eutopic endometrium are characterized by increased levels of the active form of MMP2.

We also highlighted the presence of bands at about 45 and 28 kDa, corresponding to the predicted molecular weight of the active forms of MMP3, but not at 52–55 kDa, which is the size of the pro-MMP3 (Figure 2B). Semi-quantitative analysis of the bands demonstrates a markedly increase in the abundance of both pro- and active MMP3 in endometriotic tissues, whereas healthy endometrium expresses the lowest levels of MMP3 (*p* < 0.01). Western blot analysis confirms the higher abundance of MMP10 in endometriotic lesions already shown by gene expression analysis (Figure 1). Interestingly, both OMA and DIE present relatively high levels of the active form of this metalloproteinase, compared to healthy and eutopic endometrium. In regard to TIMP1, in all analyzed tissues, the presence of a band of molecular weight of ~23 kDa corresponding to the tested protein is evident (Figure 2D). Semi-quantitative analysis of the blot demonstrated that, according to gene expression analysis, OMA has the highest TIMP1 protein level and, at the same time, reveals that TIMP1 abundance is increased in the eutopic endometrium when compared to the healthy endometrium (Figure 2D).

**Figure 3:**
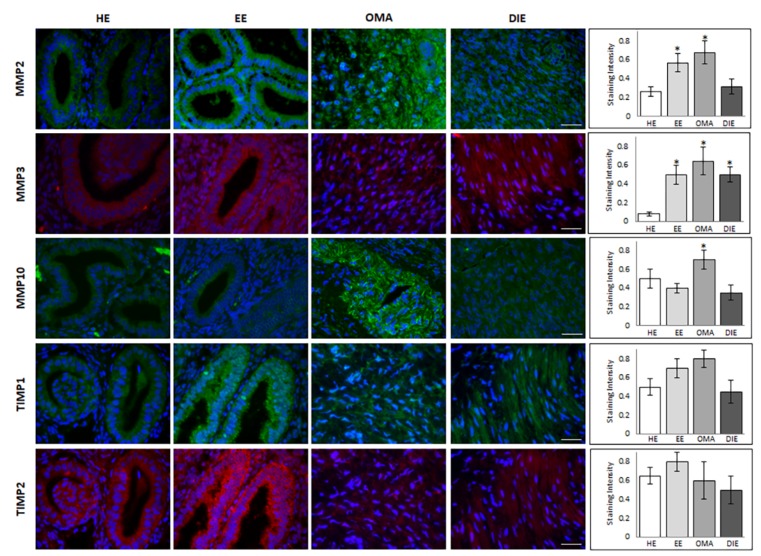
Representative images of immunofluorescence staining of MMPs and their inhibitors in healthy (HE), eutopic (EE) endometrium, in endometrioma (OMA), and in deep infiltrating lesion (DIE). MMP2 (green), MMP3 (red), MMP10 (green), TIMP1 (green), and TIMP2 (red). Nuclei were counterstained with DAPI (blue). Scale bar = 25 mm. Right histograms show the intensity of staining of MMPs and their inhibitors in HE, EE, OMA, and DIE. Data are represented as mean ± SD. * *p* < 0.05.

Finally, Western blot analysis identified a band of ~57 kDa in all analyzed samples, corresponding to the predicted molecular weight of TIMP2. Semi-quantitative analysis of the blot demonstrated the presence of increased levels of this metalloproteinase inhibitor in eutopic endometrium while very low levels may be observed in DIE. Moreover, in this case, the discrepancy with the molecular profile previously shown may reflect the high interindividual variability already highlighted in this tissue by gene expression analysis as well as the existence of pre-translational and/or post-translational modifications. Finally, in order to localize the investigated metalloproteinases and their associated tissue inhibitors, we applied immunofluorescence analysis. By this approach, MMP2 shows a prevalent epithelial glandular staining in healthy endometrium, while an intense, more diffuse staining in both the glandular and the stromal compartment may be highlighted in endometrium of patients suffering from endometriosis (Figure 3). In ovarian endometrioma, where the immunoreactivity was greater than in DIE, it is likely to observe a perinuclear localization of MMP2.

Immunofluorescence analysis not only confirmed the low abundance of MMP3 in healthy endometrium, but also demonstrated its localization as limited to the glandular epithelium; instead, an additional intense stromal staining is detectable in eutopic endometrium, as well as, even if less diffuse, in OMA and DIE lesions (Figure 3). In OMA samples, MMP10 staining is evident in both glandular and stromal compartments, with diffuse and strong signal in glands, where the intensity of MMP10 staining was the highest; the glandular localization is also evident in healthy and eutopic endometrium, while MMP10 staining appears weak and diffuse in the stromal component in DIE lesions (Figure 3).

In regard to the tissue inhibitors of metalloproteases, both TIMP1 and TIMP2 display a glandular localization in healthy and eutopic endometrium, where an intense staining shows a specific localization in the epithelial cells luminal side; by contrast, a diffuse and weak staining is evident in DIE lesions (Figure 3). In order to better compare the expression level of MMPs and their inhibitors in different cellular compartments, Supplementary Table S1 summarize the relative protein levels in glandular and stromal cellular compartments.

### 2.3. In Vitro Studies: Expression of MMPs and TIMPs in HESCs after TNFα Treatment

In order to investigate the mechanisms leading to MMPs and TIMPs deregulation in endometriotic tissues, based on the literature data reporting TNFα as a key molecule involved in establish the inflammatory environment, hallmark of endometriosis, we tested the effect of TNFα treatment on in vitro cultured endometrial stromal cells. To this end, the expression of metalloproteases and their inhibitors was assessed in healthy and eutopic HESCs treated for 48 h with 50 µM TNFα.

**Figure 4:**
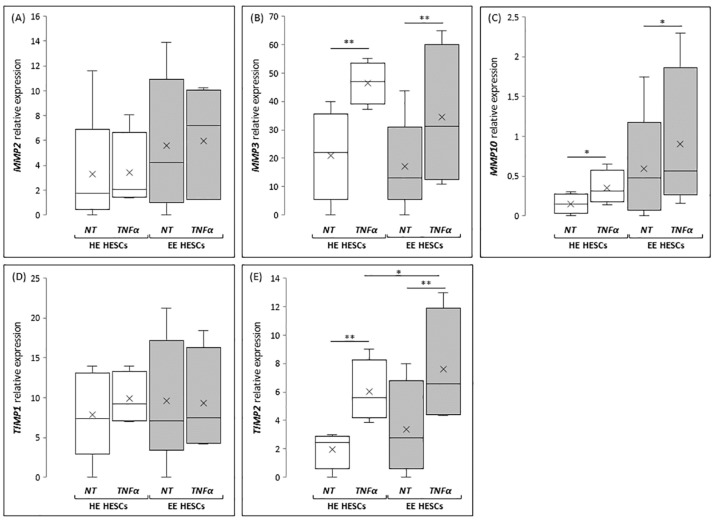
*MMP2* (**A**), *MMP3* (**B**), *MMP10* (**C**), *TIMP1* (**D**), and *TIMP2* (**E**) mRNA expression in endometrial stromal cells prepared from healthy (HE-HESCs) or eutopic (EE-HESCs) endometrium. Graphical diagram is plotted as box-whisker plots, where boxes show the interquartile range with median and mean values, and whiskers represent min and max confidence intervals. Data represent value obtained by 2^−ΔCt^ method. * *p* < 0.05; * *p* < 0.01.

According to in vivo results, the expression of *MMP2* seems to be unaffected by TNFα treatment (Figure 4A), both in HESCs prepared from healthy patients (HE-HESCs) and from women with endometriosis (EE-HESCs). By contrast, TNFα treatment is able to significantly increase *MMP3* expression both in HE-HESCs (*p* < 0.05) and EE-HESCs (*p* < 0.05) (Figure 4B). Interesting results come from the analysis of *MMP10,* whose expression was significantly increased in EE-HESCs when compared to HE-HESCs; in this case, TNFα treatment significantly increased *MMP10* expression in both cell types (*p* < 0.05 and *p* < 0.01, respectively) (Figure 4C). When the expression of inhibitors of MMPs was analyzed, no significant differences in the expression of *TIMP1* were observed, as shown in Figure 4D, whereas the expression of *TIMP2* significantly increased (*p* < 0.01; Figure 4E) in HE-HESCs treated with TNFα when compared to the untreated one. The same profile was highlighted in EE-HESCs, which after TNFα treatment showed *TIMP2* mRNA levels significantly increased not only if compared to the eutopic untreated cells (*p* < 0.01), but also when compared to HE-HESCs treated with TNFα at the same concentration (*p* < 0.05) (Figure 4).

## 3. Discussion

This study reports, for the first time, a comprehensive analysis of the overall expression of MMPs and their inhibitors in eutopic endometrium and in endometriotic lesions, in vivo as well as in vitro after treatment inducing an inflammatory response.

The main strength of the present study relies on robust inclusion criteria that are crucial for the accurate and conclusive analysis of results and thus allow a deeper understanding of MMPs and TIMPs role in endometriosis etiology. Indeed, the results of this study are normalized for uterine cycle phase, since all tissue biopsies are scheduled in proliferative phase, 2–3 days after the menstrual bleeding, thus overcoming criticisms evident in several published studies, where no attention was paid to the phase of the menstrual cycle during sample collection, which makes the results quite unreliable and difficult to analyze [24]. This criterion enabled us to avoid misinterpretation due to the bimodal distribution of MMPs and TIMPs activity during the physiological menstrual cycle, with MMPs mainly activated in the menstrual and early proliferative phases and TIMPs in the late secretory phase [24]. Another important issue comes from the systematic analysis of data through a different setup of a methodological approach including gene expression analysis, proteomic validation of data and protein localization by immunofluorescence. This made us aware of avoiding conflicting results due to the usage of different research methods having different sensitivity and specificity. For example, a significant decrease in TIMP1 levels in eutopic endometrium from women with endometriosis has been demonstrated at protein level by using enzyme-linked immunosorbent assay [25], while no statistically significant changes in TIMP1 were found when tested by quantitative polymerase chain reaction [26].

Moreover, another strength of this study was the identification of the best housekeeping genes to be used for gene expression analysis in endometrium and in endometriotic lesions. To the best of our knowledge, no data in the literature are available on the validation of suitable housekeeping genes for normalization in endometriotic tissues. These tissues display remarkable morphological and molecular differentiation; therefore, a proper normalization is strongly required in order to guarantee a precise and correct gene expression analysis.

In light of these information, here we demonstrate that different MMPs are expressed at different levels in healthy and pathological tissues, in accordance with data reported by most of the studies focused on this disease [24,25,27].

The role of MMP2 in endometriosis is still matter of debate: the available data on its expression in uterine endometrial tissue in women with and without endometriosis remains contradictory [25]. This study shows that, despite the low *MMP2* gene expression detected in eutopic endometrium, the corresponding protein seems to be upregulated. This may be due to post-transcriptional modifications or increased protein accumulation, as also suggested by several studies demonstrating that endometriotic tissues display impaired mechanisms of post-transcriptional regulation [28,29]. Moreover, this important result is in agreement with data demonstrating a higher proteolytic activity in eutopic endometrium in comparison with healthy control [25]. In this regard, we provide evidence for the presence of the active form of this metalloprotease in eutopic endometrium.

Another interesting finding of the present work is the proven increase in MMP2 expression in OMA, that confirms data reporting higher activity of MMP2 in ectopic lesions in comparison to uterine endometrium [24,30,31]. Altered expression of gelatinases has been correlated to the metastatic state; therefore, these enzymes can influence the ability of cells to invade and metastasize due to their aptitude to degrade the basement membrane [32]. This evidence could explain the increased expression of MMP2 detected in this work both in eutopic and endometriotic tissues.

As regard to stromelysins MMP3 and MMP10, their expressions significantly increased in eutopic endometrium and in endometriotic tissues, in agreement with data reported in the literature, suggesting that, like in cancer cells, stromelysin’s upregulation might contribute to the high invasive potential of endometriotic cells [26,33]. In particular, MMP3, which is known to play a significant role in ECM remodeling, is significantly overexpressed by numerous cancerous cells [34], confirming that the here reported upregulation of this protease in endometriotic tissues may be responsible for the development and progression of the disease. Despite the benign nature of this pathology (indeed for OMA, the resultant absolute risk of ovarian cancer is still low, and for DIE, the risk of developing into cancer is near zero) the recent demonstration that the majority of DIE lesions harbor cancer-associated mutations, along with the present demonstration of increased expression of protease, whose activity is significantly up-regulated in cancer, shed new light on the pathogenesis and pathophysiology of endometriosis.

According to their spectrum of activity, MMPs modulation has to be carefully controlled to prevent deleterious side effects; therefore, the importance of the regulation of their inhibitors TIMP1 and TIMP2 in reproductive tissue is evident, delineating the key role of this molecule in extracellular matrix homeostasis, the physiologic process maintaining the appropriate balance between deposition and degradation of extracellular matrix components. In this respect, this work demonstrated an increase in TIMP1 and TIMP2 expression levels in OMA. These results led us to hypothesize that an increased expression of TIMPs in these pathological tissues may be correlated with the higher expression of MMP2 and MMP3 in the same lesions as confirmed by the significant correlation demonstrated by this study. In fact, other published studies demonstrated a fine balance between the expression of MMPs and TIMPs in the processes of growth, differentiation, and destruction of tissues [27,35].

Finally, aiming to shed light on the mechanisms underlying MMP derangement in endometriotic tissues, this work underlined that TNFα treatment is able to induce a significant upregulation of *MMP10* and *TIMP2* in both healthy and eutopic HESCs. These data are relevant, since it is known that this pro-inflammatory cytokine has been implicated in the pathophysiology of endometriosis [36]. TNFα expression is increased in peritoneal fluid of women with endometriosis [37], and its levels correlate with disease severity [38], while a negative correlation has been identified between TNFα and reproductive outcomes [39,40].

Indeed, altered MMP expression is crucial for the physiology of the reproductive system, where these molecules play a crucial role in ECM remodeling during embryo implantation based on fine-tuning between MMPs activation and inhibition [41]. Impairment of this balance may induce a hostile uterine environment, characterized by endometrial inflammation, affecting a successful implantation process. Therefore, anti-inflammatory treatment, comprising of corticosteroids and antibiotics, have been proposed to reduce high MMP activity, thus improving reproductive outcomes in infertile women with repeated implantation failure [41].

Moreover, TNF-α may also play a role in the pathogenesis of endometriosis, as it has been shown that the adhesion of endometrial fibroblast to mesothelial cells is increased in vitro and specific polymorphisms in the TNFα gene are related to the predisposition to endometriosis [42].

It is well known that many MMPs and TIMPs are regulated at the transcriptional level by a variety of growth factors, cytokines, and chemokines [43,44], and TNFα is involved in the cytokine cascade for the inflammatory response that is considered a key process in pathogenesis of endometriosis [45,46]. On the other hand, MMPs are involved in inflammation through controlling the bioavailability of inflammatory mediators by regulating the integrity of the physical barriers and degrading ECM which stores inflammatory molecules. Depending on the context, the MMPs can activate, inactivate, or antagonize cytokines, which in turn can induce the synthesis of MMPs [35,47]. From this evidence, it is possible to hypothesize that the characteristic inflammatory environment of endometriosis leads to the overexpression of several MMPs which in turn release and activate various proinflammatory molecules from the ECM, leading to a self-sustained process which causes the progression of the disease.

## 4. Materials and Methods

### 4.1. Patients and Samples

For this prospective study, endometrial and endometriotic tissue samples were collected at the Obstetrics and Gynecology clinic of University Hospital of Siena, from January 2018 to October 2019. The study cohort consisted of 34 women including (i) 15 healthy women (age range between 22 and 38 years old) who underwent surgery for not endometriotic ovarian cysts and (ii) 19 endometriotic patients (age range between 21 and 40 years old) who underwent laparoscopic surgery for endometriosis. At the time of surgery, endometrial biopsy was collected from 15 healthy women and from 19 endometriotic patients; at the same time, ovarian endometrioma (OMA, *n* = 10) and deep infiltrating endometriosis (DIE, *n* = 9) lesions were collected from the 19 patients suffering from endometriosis.

A complete medical history, physical examination, and transvaginal ultrasound evaluation, carried out by the same examiner with experience in transvaginal ultrasound scans for endometriosis, was performed for each patient. According to the American Society of Reproductive Medicine (ASRM) classification (1985), all endometriosis patients enrolled in this study had stage IV of disease; this stage was histologically confirmed by pathologists. Exclusion criteria were: prior or current infections, endocrine disorders or hormonal treatment within the past 3 months and the concomitant presence of OMA and DIE lesions. All the specimens were collected during laparoscopic approach in proliferative menstrual phase, 2–3 days after the menstrual bleeding. Endometriotic specimens from OMA (*n* = 10) (cyst diameter ranging between 42 and 68 mm) were carefully stripped from the inner cyst wall and the lack of contamination with ovarian tissues was confirmed by histological evaluation. The DIE lesions (*n* = 9) were located in the posterior compartment (posterior and lateral vaginal fornices, the retrocervical area with torus uterinum and uterosacral ligaments, the parametria laterally, and the rectovaginal septum, bowel). Tissues were immediately submerged in liquid nitrogen to allow subsequent RNA and protein extraction, paraffin embedded for immunohistochemistry or immediately treated to set up primary cell cultures of HESC.

Ethical approval for the studies was obtained from the Siena University Hospital Local Ethical Committee (code 20170619, approval date 10 June 2017). All patients provided their informed consent before being enrolled in the study.

### 4.2. RNA Extraction, Complementary DNA Preparation, and Quantitative RT-PCR

Frozen samples were homogenized with TissueLyzer (Qiagen, Hilden, Germany) and RNA was extracted by using the RNeasy Mini kit (Qiagen, Hilden, Germany) according to the manufacturer’s instructions. RNA concentration was assessed using an ND-1000 Nanodrop Spectrometer (Thermo Fisher Scientific, Wilmington, DE, USA), and its integrity was evaluated by an electrophoretic run on agarose gel with FlashGel System (Lonza Group, Ltd., Basel, Switzerland). 300ng of extracted RNA were reverse transcribed into cDNA using the iScript Reverse Transcription kit (BioRad, Milan, Italy).

Gene-specific primer sets used in this study (all from Biorad Laboratories, Hercules, CA, USA) are listed in Table 1. Expression levels of *MMP2, MMP3, MMP10, TIMP1*, and *TIMP2* were determined in triplicate by qRT-PCR on a CFX connect Real-Time PCR Detection System (Bio-Rad Laboratories, Hercules, CA, USA) using SsoFast EvaGreen Supermix (Bio-Rad Laboratories, Hercules, CA, USA). The cycling conditions were as follows: 5 min at 95 °C, 30 s at 96 °C, 1 min at 58 °C, 5 min at 4 °C, and 5 min at 90 °C.

### 4.3. Western Blot

Endometrial and endometriotic tissues were suspended in RIPA^+++^ buffer for Sodium Dodecil Sulphate-polyacrylamide gel electrophoresis (SDS-PAGE). Pooled samples were obtained by mixing equal protein amounts (μg of total protein) of 5 individual samples that were extracted in the same lysis buffer. The protein concentration in pooled samples was re-estimated using the appropriate quantification protocol. About 25 ng per sample were run on 12% SDS-PAGE for electrophoresis separation and then transferred to nitrocellulose membranes in a mini Trans-Blot apparatus (Bio-Rad Laboratories, Hercules, CA, USA). Membranes were subsequently blocked with 3% *w*/*v* non-fat dry milk in 10 mMTris–HCl (pH: 7.5), 0.15 M NaCl, for 1 h at room temperature and then probed overnight at 4 °C with primary polyclonal antibody as reported in Supplementary Table S2, diluted in 1% non-fat dry milk/TTBS (TBS containing 0.2% Tween 20). After washing, membranes were incubated for 1 h with the appropriate horseradish peroxidase-conjugated secondary antibody. Immunoreactivity was detected by using chemiluminescence reagents (HRP Immuno-Star HRP substrate kit; Bio-Rad Laboratories, Hercules, CA, USA). The chemiluminescence signals were captured using a Bio-Rad Chemi-Doc system and quantified using a PDQuest analysis software (Bio-Rad Laboratories, Hercules, CA, USA).

### 4.4. Human Endometrial Stromal Cells Culture

Human endometrial stromal cells (HESCs) were isolated from healthy and eutopic endometrial biopsies immediately after collection. Tissue samples were minced into small pieces and incubated for about 2 h at 37 °C in a shaking water bath, in 10 mL of DMEM/F12 (Invitrogen) supplemented with 0.1% collagenase and DNase (0.01 mg/mL). Stromal cells were then separated by filtration with filters of 70 μm pore diameter and subsequently of 40 μm, and then they were centrifuged for 8 min at 1200 rcf and finally suspended in complete DMEM/F12 medium supplemented with 10% charcoal-stripped calf serum (Sigma-Aldrich). The lower passage number (P0–P4) of cells was used for experiments to avoid changes in phenotype and gene expression. The medium was changed to a serum-free DMEM/F12, 24 h before each treatment, performed in duplicate. To determine the effect of TNFα on gene expression, the cells were stimulated for 48 h with recombinant human TNFα (Peprotech, London, UK) at a final concentration of 20µM or with free medium alone (control condition). Cells were collected with 350 µL RLT and stored at −80 °C until RNA extraction.

### 4.5. Immunofluorescence

For immunofluorescence analysis, cells were grown on coverslips, washed with phosphate-buffered saline solution (PBS), and fixed for 15 min in 4% paraformaldehyde and then permeabilized in 0.5% Triton X-100 in PBS for 10 min. Coverslip slides were immunolabeled using an indirect procedure. After blocking in 5% BSA in PBS for 30 min, cells were incubated 1 h at 25 °C with the primary antibodies (see Supplementary Table S2), washed three times with PBS, and then incubated for 1 h at room temperature with the secondary antibodies (see Supplementary Table S2). Nuclei were stained with 6-diamino-2-phenylindole in mounting medium (Santa Cruz Biotechnology). After washing in PBS, the slides were mounted with an antifade solution, in order to avoid fading of fluorescence, and immediately observed with fluorescence Leica microscope equipped with LAS software. Fluorescence images were acquired and stored in JPEG format for subsequent staining quantification analysis, performed by using both LAS and Image J software. In order to quantify total fluorescence staining, for each samples, at least 10 fields were analyzed with LSA and Image J software, by two independent observers. In order to measure fluorescence staining in stromal and glandular compartments, regions of interest (ROIs) were drawn manually in the whole slide overview of the image section. The quality of the tissue and the staining efficacy in each slide has been estimated by the DAPI staining pattern of nuclei.

### 4.6. Statistical Analysis

Statistical analysis was performed using the GraphPad Prism 5.0 (GraphPad Software, San Diego, CA, USA). Statistical significance was evaluated by using nonparametric tests. Differences among groups of data were tested by the Kruskal–Wallis test or the Mann–Whitney test. Statistical significance was set at *p* < 0.05. Correlation was determined by using Spearman’s correlation analysis.

## 5. Conclusions

The systematic methodological approach set up in this study allows to overcome the conflicting results still present in the literature, thus clarifying the pivotal role of MMPs and their inhibitors in this pathology. Indeed, although further studies are needed to fully understand the molecular mechanisms involved in these processes, the present data confirm that investigated MMPs and their inhibitors are altered both in eutopic endometrium and endometriotic lesions. These findings demonstrate that the deregulation of ECM remodeling is a crucial process for the endometrial physiology, and it may lead to an increase in the ability of endometrial cells to implant and to originate an invasive growth of the endometrial tissue. We would like to disclose that, at this time, the data reported in this study does not allow the use of endometrial expression of MMPs and their inhibitors expression in the diagnostic process of endometriosis. Indeed, further research on the role of MMPs and TIMPs in the onset and development of the endometriosis in a larger study groups would be required before clinical application. Based on our preliminary data, we may hypothesize that the timely detection and the fine-tuning of endometrial MMPs activity should be effective in endometriosis diagnosis and management, thus providing new targets for the clinical treatment of endometriosis.

## Figures and Tables

**Table 1:** Primers for qRT-Polymerase Chain Reaction.

**Target Genes**	**Acronym**	**ID Assay**
*Matrix metalloproteinase-2*	**MMP2**	qHsaCID0015623
*Matrix metalloproteinase-3*	**MMP3**	qHsaCID0006170
*Matrix metalloproteinase-10*	**MMP10**	qHsaCID0008481
*Tissue inhibitor of metalloproteinases-1*	**TIMP1**	qHsaCID0007434
*Tissue inhibitor of metalloproteinases-2*	**TIMP2**	qHsaCID0022953
**Reference Genes**	**Acronym**	**ID Assay**
*Actin, beta*	**ACT-B**	qHsaCED0036269
*Beta-2-microglobulin*	**B2M**	qHsaCID0015347
*Glyceraldehyde-3-phosphate dehydrogenase*	**GAPDH**	qHsaCED0038674
*Hypoxanthine phosphoribosyltransferase 1*	**HPRT1**	qHsaCID0016375
*Peptidylprolyl isomerase B (cyclophilin B)*	**PPIB**	qHsaCID0016928
*TATA-box binding protein*	**TBP**	qHsaCED0044763

To select the more appropriate calibrator gene, the expression levels obtained from each sample of six reference genes were analyzed with the use of two different VisualBasic (VBA) applets, geNorm (integrated in the qbase software at: https://www.qbaseplus.com). The reference genes were then used to normalize mRNA amount, obtaining a final value of relative gene expression.

## Supplementary Materials

The following are available online at https://www.mdpi.com/1422-0067/21/8/2840/s1, Supplementary Table S1: HeatMap for relative protein abundance of MMPs and TIMPs in glandular and stromal compartments, measured by immunofluorescence; Supplementary Table S2: List of antibodies used in this study.
