# Supplementary material for: Expression of Matrix Metalloproteinases and Their Inhibitors in Endometrium: High Levels in Endometriotic Lesions
Source: Int J Mol Sci. 2020 Apr 18;21(8):2840. doi: 10.3390/ijms21082840 (PMC7215833; doi:10.3390/ijms21082840)
Supplement: Supplementary file 1 [file ijms-21-02840-s001.pdf]

**Supplementary Table S1:** HeatMap for relative protein abundance of MMPs and TIMPs in glandular and stromal compartments, measured by immunofluorescence.

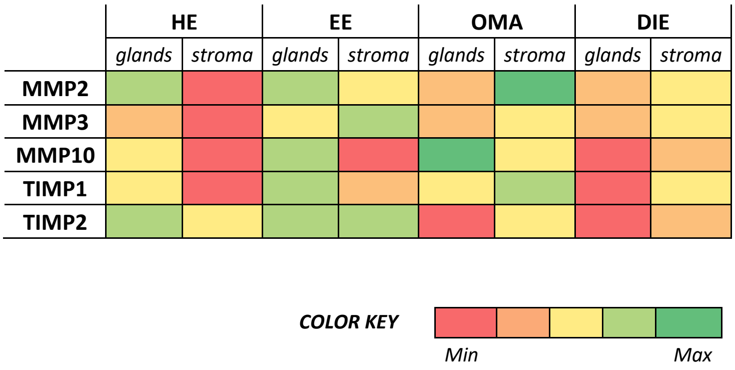


**Supplementary Table S2.** List of antibodies used in this study

| *Antigen* | *RRID* | *Donor species* | *Dilution* | |
|---|---|---|---|---|
| | | | *IF* | *WB* |
| ***Primary antibodies*** | | | | |
| ACT-B | AB_306374 | Goat | / | 1:500 |
| MMP2 | AB_10696122 | Rabbit | 1:200 | 1:500 |
| MMP3 | AB_303604 | Mouse | 1:200 | 1:500 |
| MMP10 | AB_944225 | Rabbit | 1:200 | 1:500 |
| TIMP1 | AB_10864175 | Rabbit | 1:200 | 1:300 |
| TIMP2 | AB_10740374 | Mouse | 1:100 | 1:200 |
| ***Secondary antibodies*** | | | | |
| Anti-Rabbit-IgG FITC | AB_259430 | Goat | 1:500 | 1:100 |
| Anti-Mouse IgG AlexaFluor 546C | AB_2534085 | Goat | 1:500 | 1:100 |
| Anti-Goat-IgG HRP | AB_92411 | Rabbit | / | 1:7500 |
| Anti-Rabbit-IgG HRP | AB_11212848 | Goat | / | 1:2000 |
| Anti-Mouse-IgG HRP | AB_955440 | Rabbit | / | 1:3000 |
